# Non-Clinical In Vitro Evaluation of Antibiotic Resistance Gene-Free Plasmids Encoding Human or Murine IL-12 Intended for First-in-Human Clinical Study

**DOI:** 10.3390/pharmaceutics13101739

**Published:** 2021-10-19

**Authors:** Spela Kos, Masa Bosnjak, Tanja Jesenko, Bostjan Markelc, Urska Kamensek, Katarina Znidar, Urska Matkovic, Andrej Rencelj, Gregor Sersa, Rosana Hudej, Aneja Tuljak, Matjaz Peterka, Maja Cemazar

**Affiliations:** 1 Department of Experimental Oncology, Institute of Oncology Ljubljana, Zaloska cesta 2, SI-1000 Ljubljana, Slovenia; skos@onko-i.si (S.K.); mbosnjak@onko-i.si (M.B.); tjesenko@onko-i.si (T.J.); bmarkelc@onko-i.si (B.M.); ukamensek@onko-i.si (U.K.); kznidar@onko-i.si (K.Z.); umatkovic@onko-i.si (U.M.); arencelj@onko-i.si (A.R.); gsersa@onko-i.si (G.S.); 2 Faculty of Pharmacy, University of Ljubljana, Aškerceva ulica 7, SI-1000 Ljubljana, Slovenia; 3 Faculty of Medicine, University of Ljubljana, Vrazov trg 2, SI-1000 Ljubljana, Slovenia; 4 Faculty of Health Sciences, University of Ljubljana, Zdravstvena pot 5, SI-1000 Ljubljana, Slovenia; 5 Biotechnical Faculty, University of Ljubljana, Jamnikarjeva ulica 101, SI-1000 Ljubljana, Slovenia; 6 Center Odličnosti za Biosenzoriko, Instrumentacijo in Procesno Kontrolo, Mirce 21, SI-5270 Ajdovscina, Slovenia; rosana.hudej@cobik.si (R.H.); aneja.tuljak@cobik.si (A.T.); matjaz.peterka@cobik.si (M.P.); 7 Faculty of Health Sciences, University of Primorska, Polje 42, SI-6310 Izola, Slovenia

**Keywords:** interleukin 12, gene electrotransfer, antibiotic resistance gene-free plasmid, non-clinical evaluation

## Abstract

Interleukin 12 (IL-12) is a key cytokine that mediates antitumor activity of immune cells. To fulfill its clinical potential, the development is focused on localized delivery systems, such as gene electrotransfer, which can provide localized delivery of IL-12 to the tumor microenvironment. Gene electrotransfer of the plasmid encoding human IL-12 is already in clinical trials in USA, demonstrating positive results in the treatment of melanoma patients. To comply with EU regulatory requirements for clinical application, which recommend the use of antibiotic resistance gene-free plasmids, we constructed and developed the production process for the clinical grade quality antibiotic resistance gene-free plasmid encoding human IL-12 (p21-hIL-12-ORT) and its ortholog encoding murine IL-12 (p21-mIL-12-ORT). To demonstrate the suitability of the p21-hIL-12-ORT or p21-mIL-12-ORT plasmid for the first-in-human clinical trial, the biological activity of the expressed transgene, its level of expression and plasmid copy number were determined in vitro in the human squamous cell carcinoma cell line FaDu and the murine colon carcinoma cell line CT26. The results of the non-clinical evaluation in vitro set the basis for further in vivo testing and evaluation of antitumor activity of therapeutic molecules in murine models as well as provide crucial data for further clinical trials of the constructed antibiotic resistance gene-free plasmid in humans.

## 1. Introduction

Interleukin 12 (IL-12) is one of the most potent proinflammatory cytokines in the mediation of the antitumor activity of immune cells. It has long been studied as a potential immunotherapeutic for cancer based on its ability to engage multiple effector mechanisms and reverse tumor-induced immunosuppression [1]. Among its pleiotropic effects, IL-12 induces Th1 cell differentiation, increases activation and cytotoxicity of T lymphocytes and natural killer (NK) cells and inhibits immunosuppressive cells, such as tumor-associated macrophages or myeloid-derived suppressor cells [1]. Most of the IL-12-induced effects are mediated by the secretion of interferon gamma (IFNγ), which itself exerts cytostatic and antiangiogenic activities and upregulates the major histocompatibility complex (MHC) I and MHC II expression on tumor cells for enhanced recognition and lysis. Consequently, IL-12 has demonstrated a significant antitumor activity against a wide range of malignancies in preclinical models and a strong immunostimulatory potential in humans. Nevertheless, despite the encouraging results of the IL-12 therapy, systemic administrations of recombinant IL-12 incurs exceeding toxicity. To overcome the limitations of systemic delivery, novel delivery techniques have emerged, which provide more effective and less toxic IL-12 delivery to the tumor microenvironment. One of them is electrically mediated gene delivery of the plasmid coding for IL-12. Gene electrotransfer (GET) is a well-established electroporation-based gene delivery method that can be used to deliver plasmid DNA into target cells or tissues. Among the non-viral delivery methods, GET is considered to be one of the more efficient, low-cost and reproducible [2]. As a therapeutic application, GET has mostly been used as DNA vaccination for infectious diseases and in gene therapy for the treatment of cancer [3,4].

The first studies involving GET of the plasmid coding for IL-12 (GET IL-12) were performed on healthy non-tumor-bearing mice, evaluating the effect of GET IL-12 after transfection of the skin and muscles [5,6]. The efficiency of GET IL-12 was successfully demonstrated by increased systemic concentrations of IL-12 and its effector molecule IFNγ. Furthermore, the antitumor effectiveness of GET IL-12 was confirmed with intratumoral injections of plasmid DNA encoding IL-12 delivered by GET [7], resulting in significant inhibition of the growth of treated tumors and, additionally, the growth of untreated distant metastases. Afterwards, the feasibility of GET IL-12 was further tested in various tumor models, such as models of murine squamous cell carcinoma, melanoma, colorectal carcinoma, kidney cancer, lymphoma, breast carcinoma and sarcoma [8].

Preclinical studies on murine tumor models have shown that GET IL-12 is specifically successful in the treatment of skin tumors and their metastases [8]. Therefore, due to its remarkable antitumor activity at the preclinical level, GET IL-12 has progressed to a number of clinical trials in both human and veterinary medicine, mainly for the treatment of skin cancers. Several canine clinical studies have explored GET IL-12 in companion dogs with naturally occurring tumors [1]. In one such study, GET IL-12 resulted in a notable reduction in the mast cell tumor volume, with increased inflammatory cell infiltration of the treated tumors and a decreased number of malignant mast cells [9]. Another study investigated GET IL-12 in the combination with electrochemotherapy and cytoreductive surgery for the treatment of canine oral malignant melanoma [10]. The therapy resulted in a 67% objective response rate accompanied by a decrease in the percentage of regulatory T cells in the peripheral blood. The effects were ascribed to a systemic antitumor response to GET IL-12.

Several human clinical trials in the USA, mainly against advanced melanoma, have addressed the safety and efficiency of GET IL-12. The first clinical study on GET IL-12 was published in 2008. The study included 24 patients with skin metastatic melanoma. The plasmid encoding human IL-12 under the control of the cytomegalovirus (CMV) promoter and with a gene for resistance to kanamycin as the selection gene was used. Gene therapy was performed three times on each tumor, resulting in a good local clinical response of the treated tumors and in a systemic antitumor effect on the distant non-treated nodules in 53% of the patients [11]. In a Phase II study, where 29 melanoma patients were treated, an objective response rate of 33% with 11% complete responses was achieved. The treatment was found to increase the presence of NK cells both intratumorally and systemically, induce a systemic T cell response and recruit T cells to the tumor microenvironment [12]. The treatment was well-tolerated without any observed serious adverse effects.

Despite the encouraging results, the plasmids used in the described studies contain an antibiotic resistance gene serving as a selection marker for the production of plasmid DNA. The presence of antibiotic resistance genes raises safety concerns, often pointed out by the regulatory authorities [13]. To comply with the European Union’s regulatory requirements for clinical application, in our previous study, a human IL-12-encoding plasmid devoid of an antibiotic resistance gene (p21-hIL-12-ORT, phIL12) was constructed [14]. Additionally, to further improve the safety profile and achieve a more controllable expression, the promoter driving the expression of IL-12 was switched from a constitutive one to the tumor-specific and genotoxic stress-inducible promoter of the cyclin-dependent kinase inhibitor 1 (CDKN1a) gene, more commonly known as p21 [14,15,16,17,18,19]. An advantage of this promotor is that, due to its endogenous origin, it is not prone to transcriptional inactivation, as it is often the case with the virus-derived promoters [20]. The promoter provides low basal expression in normal cells and high basal expression in the tumor cells. Expression can be further increased by different treatment-induced stresses, making it ideal for a combinational therapy with a conventional cancer treatment such as radiotherapy or chemotherapy planned in the future. For this study, a murine ortholog of the phIL12 plasmid was also constructed since human IL-12 is not active in mice (p21-mIL-12-ORT, pmIL12).

Plasmid DNA is generally prepared from *Escherichia coli* cultures followed by cell lysis to release the plasmid from cells and different purification strategies depending on purity requirements. The main challenge is to design a scalable, robust and reproducible manufacturing process, resulting in a product meeting quality standards. The plasmid DNA product must be of high purity, essentially in its supercoiled form, and free of host cell proteins, chromosomal DNA, RNA and endotoxins. Unit operations are well-known and were described previously in detail [21,22].

According to the European Medical Agency (EMA) guideline on the quality, non-clinical and clinical aspects of gene therapy medicinal products (EMA/CAT/80183/2014) and the International Council for Harmonisation of Technical Requirements for Pharmaceuticals for Human Use (ICH) guideline Q6B “Specifications: Test Procedures and Acceptance Criteria for Biotechnological/Biological Products” (CPMP/ICH/365/96), acceptance criteria for drug substances and drug products are not predefined. Acceptance criteria should be established and justified based on the data obtained from the lots used in preclinical and/or clinical studies. Extensive characterization should be performed in the development phase while also following significant process changes. The United States Pharmacopeia, on the other hand, clearly defines specifications for plasmid DNA drug products.

The aim of this study was to develop plasmid production in a clinical grade quality process and evaluate the in vitro effects of the phIL12 and pmIL12 plasmids in the human squamous cell carcinoma cell line FaDu and the murine colon carcinoma cell line CT26, respectively, according to the guidelines for non-clinical testing of medicines. Therefore, we aimed to evaluate the level of transgene expression on the mRNA and protein levels, the biological activity of the produced proteins and the plasmid copy number in the targeted cells. The results of the study set the basis for in vivo testing and evaluating antitumor activity of therapeutic molecules in murine models as well as provide the necessary data for clinical trials using the constructed antibiotic resistance gene-free plasmids for the treatment of skin tumors in cancer patients.

## 2. Materials and Methods

**Figure 1:**
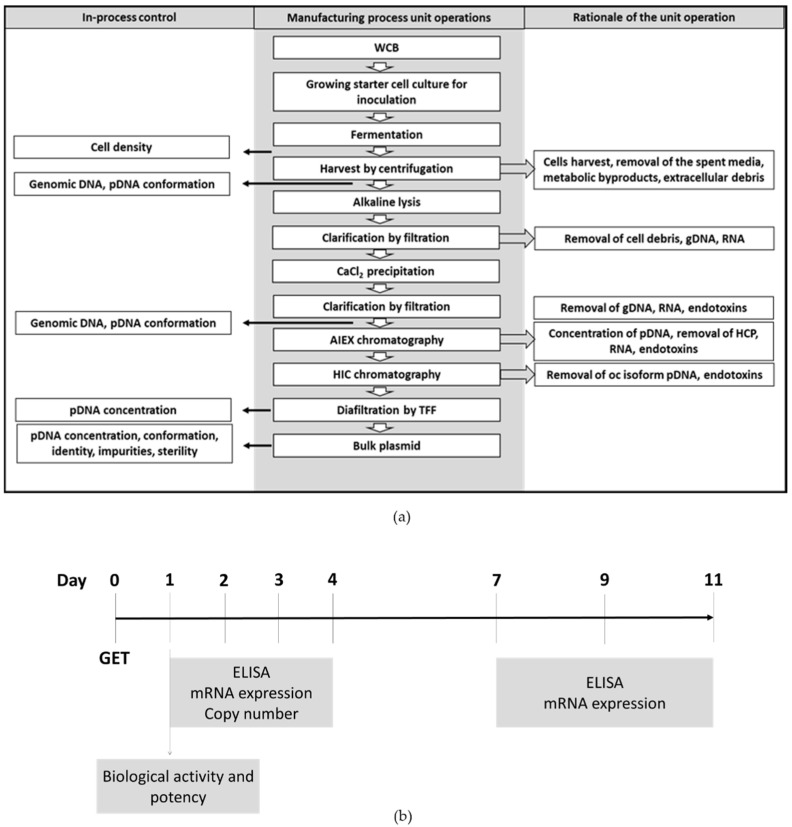
Designated study protocols. (**a**) Manufacturing process flowchart for phIL12 and pmIL12 production with unit operations and the in-process control sequence. (**b**) Timeline of the in vitro experiments.

The manufacturing process was developed with the goal of producing plasmid DNA that meets the appropriate quality specifications to enable initiation of a clinical trial. The production flowchart in Figure 1a shows the main stages of the manufacturing process for the phIL12 and pmIL12 plasmids including the in-process controls for quality control check.

Fermentation in a bioreactor was performed in a defined animal component-free medium while all the reagents used in the upstream and downstream processing were current good manufacturing practice (cGMP)-compliant. The critical steps in the process that strongly influence plasmid quality and purity were defined and closely monitored through the in-process control as recommended by the regulatory guidelines.

The production process for clinical grade plasmids was established for phIL12 and further optimized for pmIL12 production. Special emphasis was placed on robustness of the production process which will enable production of large quantities of clinical grade plasmids for future clinical studies. The acceptance criteria for drug products will be established based on the lots used for non-clinical evaluation.

For the in vitro evaluation of plasmid phIL12, the experiments were performed on the human squamous cell carcinoma cell line FaDu, which is a well-characterized commercially available human head and neck cell line. The FaDu cell line is the commercially available cancer cell line that best resembles tumors to be treated in the proposed clinical trial, which is basal cell carcinoma of the skin. However, due to the biological inactivity of human IL-12 in mice, we substituted phIL12 with pmIL12, a plasmid DNA encoding murine interleukin 12 (p21-mIL-12-ORT). As a model cell line for the in vivo experiments, we selected a murine colon carcinoma CT26 tumor cell line due to the lack of commercially available murine basal cell carcinoma cell lines. To set the basis for further in vivo experiments, all the in vitro experiments performed on the FaDu cell line were additionally performed on the CT26 cell line. Thus, the in vitro studies were performed to determine the biological activity of both phIL12 and pmIL12, their potency, the level of transgene expression, the plasmid copy number and the maintenance of the sequence in the transfected cell lines 11 days after transfection (Figure 1b).

The protocol for the evaluation of the prepared plasmids was planned in accordance with the following EMA guidelines: EMA/CAT/80183/2014, EMA/CAT/852602/2018, EMEA/CHMP/GTWP/125459/2006, EMA/CPMP/ICH/286/1995, EMA/CHMP/ICH/646107/2008, EMEA/273974/2005, CPMP/BWP/3088/99, CPMP/SWP/1042/99 (rev. 1, corr.) and reflection paper “Expectations for Biodistribution (BD) Assessments for Gene Therapy (GT) Products”. The phIL12 and pmIL12 used to perform the non-clinical evaluation had the same specifications as the phIL12 that will be used in clinical trials.

### 2.1. Plasmids Construction and Master Cell Bank (MCB) Generation

The plasmids were prepared using the conventional molecular cloning methods of restriction and ligation coupled with the operator–repressor titration (ORT) technology that enables the construction of antibiotic resistance gene-free plasmids. All the reagents and kits used for cloning (GeneJET Plasmid Miniprep Kit, TransformAid Bacterial Transformation Kit supplied with *E. coli* strain JM109, FastDigest Restriction Enzymes, Rapid DNA Ligation Kit, GeneJET Gel Extraction Kit, Sybr Safe and Sybr Gold Gel Stain) were purchased from Thermo Fisher Scientific, Waltham, MA, USA. The ORT technology using DH1-PEPA and DH1-ORT *E. coli* cells (Cobra Biologics, Keele, UK) was used to prepare the final antibiotic resistance gene-free plasmid. The SnapGene software (GSL Biotech LLC, San Diego, CA, USA) was used for sequence assembly, cloning planning and simulation of agarose gel electrophoresis. The murine and human IL-12 fusion genes (p40 and p35 subunits) originated from the commercially available plasmids: human—from the pORF-hIL-12 G2 plasmid, murine—from pORF-mIL-12 (p40:p35) (both: InvivoGen, Toulouse, France). The coding sequence for the p21 promoter originated from the WWP-Luc plasmid, which was a gift from Bert Vogelstein (Addgene, Watertown, MA, USA; Adgene plasmid No. 16451, www.addgene.org/16451/) [23]. Additionally, the pCR-blunt psiCAT plasmid (Cobra Biologics) was used as a vector to prepare the antibiotic resistance gene-free plasmids.

**Figure 2:**
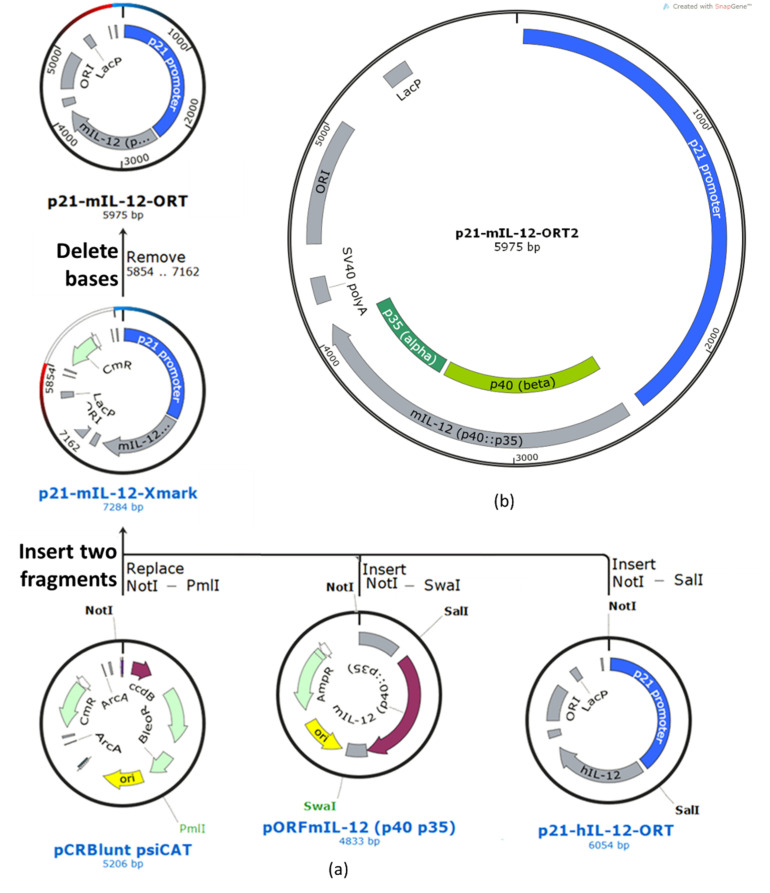

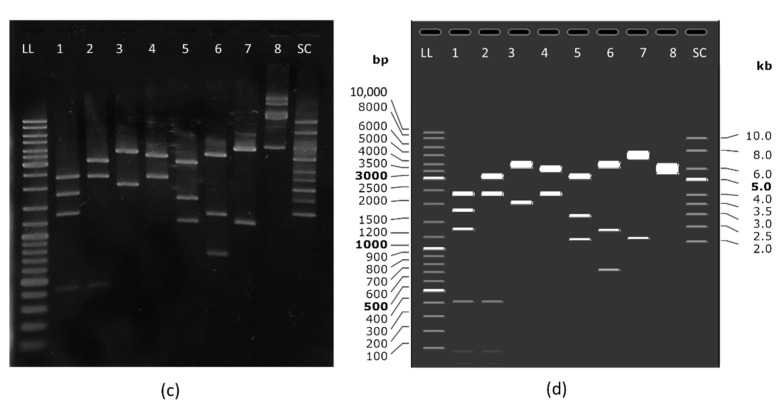
Construction and confirmation of the p21-mIL-12-ORT plasmid. (**a**) Cloning plan created using the SnapGene software: the expression cassette carrying the murine IL-12 sequence under the EF1/HTLV promoter was cut out of the pORF-mIL-12 (p40:p35) plasmid with the *Not*I and *Swa*I (blunt end) restriction enzymes and ligated to the pCR-blunt psiCAT plasmid cut with *Not*I and *Pml*I (blunt end). In the resulting plasmid, the promoter region was replaced with the p21 promoter from the p21-hIL-12-ORT plasmid using the *Not*I and *Sal*I restriction enzymes. The chloramphenicol antibiotic resistance gene (CmR) was then removed from the p21-mIL-12-Xmark plasmid using the ORT technology, resulting in the p21-mIL-12-ORT plasmid. (**b**) Annotated plasmid map: p21 promoter–promoter region from the native human p21 (CDKN1A) gene, mIL-12 (p40:p35) murine IL-12 intronless open reading frame consisting of the IL-12b (p40, beta subunit) and IL-12a (p35, alpha subunit) genes, SV40 polyA-simian virus 40 late polyadenylation signal, ORI-*E. coli* origin of replication, LacP lactose operon promoter with the lacO operator. (**c**,**d**) Restriction analysis: (**c**) the plasmid was cut with different combinations of restriction enzymes and its identity was confirmed by positive matching of the pattern of bands on the electrophoresis gel to the expected (**d**) pattern obtained by means of a simulation experiment using the SnapGene software. For the uncut plasmid, the simulated band pattern differs from the actual pattern because simulation can only be done for a supercoiled monomer, while other forms (supercoiled dimer, open circular, linear, nicked) can also be seen on the electrophoretic gel. Electrophoresis details: 1% agarose (Sigma-Aldrich), run for 45 min at 100 V/cm, stained in 1× Sybr Gold (Thermo Fisher Scientific). LL (linear DNA ladder): GeneRuler™ 1 kb Plus DNA Ladder (Thermo Fisher Scientific), lane 1: HindIII + MunI (2338 bp, 1792 bp, 1338 bp, 409 bp, 79 bp, 19 bp), lane 2: HindIII (3130 bp, 2338 bp, 409 bp, 79 bp, 19 bp), lane 3: KpnI (3936 bp, 2039 bp), lane 4: NcoI + Alw44I (3633 bp, 2342 bp), lane 5: BamHI + XbaI (3150 bp, 1652 bp, 1173 bp), lane 6: KpnI + Alw44I (3936 bp, 1314 bp, 725 bp), lane 7: NotI + Alw44I (4787 bp, 1188 bp), lane 8: uncut (supercoiled 5975 bp), SC (supercoiled DNA ladder): Supercoiled DNA Ladder (New England BioLabs, Ipswich, MA, USA).

The construction of the p21-hIL-12-ORT plasmid was described in our previous paper [14]. To prepare the murine ortholog p21-mIL-12-ORT, the p21 sequence from the p21-hIL-12-ORT plasmid was ligated to the murine IL-12 gene from the pORF-mIL-12 (p40:p35) plasmid and cloned to the pCR-blunt psiCAT plasmid. The resulting recombinant plasmid with the murine IL-12 fusion gene under the transcriptional control of the p21 promoter (still containing the antibiotic resistance gene) was transformed into the DH1-PEPA *E. coli* cells, and the transformed bacteria were selected on selective LB agar plates with chloramphenicol (Merck Millipore, Burlington, MA, USA). To confirm the presence of the transformed plasmid, miniprep isolations were performed, and the clones were confirmed using restriction analysis. The aliquots of the confirmed clone were stored in 20% glycerol at −80 °C for future use. The isolated plasmid was further transformed into the DH1-ORT *E. coli* cells, in which the antibiotic resistance gene was excised by Xer recombination [24]. Transformation was followed by selection of the clones that contained the antibiotic resistance gene-free version of the plasmid by replica-streaking on gridded agar plates with and without the selection antibiotic. From the positive clones, a single clone was selected that gave the highest yields according to the miniprep isolations and the highest supercoiled ratio according to electrophoretic separation on agarose gel (Supplementary Figure S1). The selected clone was confirmed by restriction analysis. Finally, the plasmid was confirmed by full-length plasmid sequencing and an annotated plasmid map was created based on the sequencing results (Figure 2).

The MCB was prepared by means of isolation of a single colony that was grown in 50 mL LB Broth (Sigma-Aldrich, St. Louis, MO, USA) at 37 °C in a 250 mL shaker flask at 180 rpm. When an OD600 of 0.6 was reached, glycerol (Sigma-Aldrich) was added to the culture (20% *v*/*v*), and aliquots of 1 mL were frozen at −80 °C, forming the MCB. Plasmid identity was confirmed by restriction analysis.

### 2.2. Production of Plasmids of Clinical Grade Quality

Both plasmids, p21-hIL-12-ORT and p21-mIL-12-ORT, were produced using the identical manufacturing process steps.

#### 2.2.1. Fermentation

The inoculum of the fermenter consisted of a shake flask culture. One vial of the MCB was transferred into a shake flask containing 100 mL of a defined medium consisting of a C-source glucose (Sigma-Aldrich), a macroelements solution and a trace elements solution. The culture was grown for approximately 16 h at 30 °C and 200 rpm. This culture was inoculated into a Minifors 2 benchtop fermenter with a working volume of 3 L (Infors HT, Bottmingen, Switzerland) containing 1000 mL of the defined medium with the EX-CELL or Poly (propylene glycol) antifoaming agent (both: Sigma-Aldrich). During fermentation, pH was controlled at 7.0 with 10% NH_4_OH (Honeywell, Charlotte, NC, USA). Air inflow in the range of 0.1–1.5 L/min and agitation speed in the range of 300–1000 rpm were automatically feedback-controlled based on dissolved oxygen (DO) at a set point of 20%.

#### 2.2.2. Harvest and Lysis

The cells were harvested using a custom-made tangential flow filtration system equipped with a hollow fiber 500 kDa cartridge with a membrane area of 420 cm^2^ (GE Healthcare, Chicago, IL, USA). The cells were concentrated from 2.5 L to 0.5 L. The harvest pool was exchanged for sterile buffer A (50 mM Tris HCl, pH 8.0, 10 mM EDTA). Lysis was performed at room temperature by adding 1.0 L of sterile buffer B (1% SDS, 0.2 M NaOH) and mixing. Five min after lysis, the cellular debris, the genomic DNA (gDNA) and the proteins were precipitated by gently adding and mixing 0.5 L of cooled (4–10 °C) buffer C (3 M potassium acetate, pH 5.5). After 3 min of mixing, the lysate was filtered through Sartoclear^®^ Maxicap^®^ 5” (Sartorius, Göttingen, Germany). The cleared lysate was adjusted to 0.75 M CaCl_2_ by adding 4 M CaCl_2_ and stored at 2–8 °C for 15 min followed by filtration through Sartopore^®^ 2 0.45 μm, MidiCaps (Sartorius).

#### 2.2.3. Purification

Plasmid purification was performed by means of two-step purification on anion exchange and hydrophobic interaction chromatography columns. The cleared lysate was adjusted to 40.0 ± 2.0 mS/cm and loaded on CIMmultus™ DEAE–8 mL (BIA Separations, Ajdovscina, Slovenia) equilibrated with 200 mM Tris, 10 mM EDTA, pH 8.0. Plasmid DNA was separated from the host cell protein and RNA in step gradient of NaCl and eluted with 200 mM Tris, 10 mM EDTA, 1.0 M NaCl, pH 8.0. The eluted fraction containing plasmid DNA was adjusted to 3.0 M ammonium sulphate (AS, Merck Millipore) and loaded on a CIMmultus™ C4 HLD–8 mL (BIA Separations) column equilibrated with 50 mM Tris, 10 mM EDTA, 2.0 M AS, pH 7.2. Supercoiled plasmid DNA (Sc) was separated from the open circular plasmid DNA (Oc) and gDNA in step gradient of AS and eluted with 50 mM Tris, 10 mM EDTA, 1.0 M AS, pH 7.2 [25]. The eluted fraction containing plasmid DNA was exchanged to 0.9% NaCl with diafiltration on Pellicon^®^ XL, Ultracel 30 with a membrane area of 50 cm^2^ (Merck Millipore). In the p21-hIL-12-ORT production process, diafiltration consisted of two repetitions (10-fold dilution and concentration), while three repetitions were performed in the p21-mIL-12-ORT production process. Finally, the solution was filtered through a 0.22 μm filter, aliquoted to cryovials and stored at −80 °C until use.

#### 2.2.4. Plasmid DNA Qualification

Qualification of pDNA was carried out using an in-house developed method on a high-performance liquid chromatography (HPLC) system Prominence (Shimadzu, Kyoto, Japan) and a CIMac™ pDNA-0.3 Analytical Column (BIA Separations). Twenty μL of the sample containing plasmid DNA were injected at a flow rate of 1 mL/min. As the equilibration buffer, 200 mM Tris (pH 8.0) were used. Elution of plasmid DNA and impurities was achieved by applying the linear gradient of the elution buffer (200 mM Tris, 1.0 M NaCl, pH 8.0). Detection was carried out at a wavelength of 260 nm.

#### 2.2.5. Host Cell Protein Quantification

A specialized bicinchoninic acid assay version for low protein concentrations Micro BCA Protein Assay Kit from Thermo Fischer Scientific (Waltham, MA, USA) was used to measure the residual protein impurities in accordance with the manufacturer’s instructions. The calibration curve with seven different concentrations including the blank was constructed with the BSA protein standard, and the absorbance at 562 nm was measured in triplicates on a Synergy H1 Hybrid Reader (BioTek Instruments, Winooski, VT, USA). The absorbance for the plasmid samples was measured in duplicates with additional duplicates for spike recovery calculations. The acceptance criteria for spike recovery were determined in order to avoid erroneous results. The averaged values were used to form a standard curve from which the host cell protein concentration in the plasmid sample was calculated.

### 2.3. Cell Cultures

The human squamous cell carcinoma cell line FaDu (obtained from ATCC; ATCC^®^ HTB43™) and the murine colon carcinoma CT26.WT (hereinafter referred to as CT26) tumor cell line (obtained from ATCC, Manassas, VA, USA; ATCC^®^ CRL-2638™) were cultured in the ATCC-suggested cell culture mediums. The FaDu cells were cultured in Advanced Dulbecco’s Modified Eagle’s Medium (A-DMEM, Gibco, Thermo Fisher Scientific, Waltham, MA, USA) and the CT26 cells were cultured in Advanced RPMI-1640 (A-RPMI, Gibco), both supplemented with 2 mM L-glutamine (Gibco), 5% (*v*/*v*) fetal bovine serum (FBS, Gibco), GlutaMAX (Gibco) and 1% (*v*/*v*) Penicillin–Streptomycin (stock solution, 10,000 U/mL, Gibco). Additionally, HEK-Blue™ IL-12 cells (IL-12 reporter cells) (InvivoGen) were used to determine the biological activity and potency of phIL12 and pmIL12. The HEK-Blue™ IL-12 cells were cultured in A-DMEM (Gibco) supplemented with 2 mM L-glutamine (Gibco), 5% (*v*/*v*) FBS (Gibco) and 1% (*v*/*v*) Penicillin–Streptomycin (stock solution, 10,000 U/mL, Gibco). After the second passage, the 1× HEK-Blue™ Selection (InvivoGen) was added to the growth medium. The cells were handled according to the supplier’s instructions and cultured in a 5% CO_2_ humidified incubator at 37 °C. For the experiments, the cells were maintained in monolayers until they reached 70–80% confluence.

### 2.4. Gene Electrotransfer (GET)

In the proposed clinical trial, electroporation, i.e., GET, will be used to transfect tumors with phIL12. Therefore, electroporation was also used to transfect the cells in vitro. For this purpose, a suspension of the FaDu or CT26 cells in the exponential growth phase was trypsinized and prepared in a cold electroporation buffer (EP buffer; 125 mM sucrose, 10 mM K_2_HPO_4_, 2.5 mM KH_2_PO_4_, 2 mM MgCl_2_ × 6 H_2_O) with a concentration of 25 × 10^6^ cells/mL. Then, 40 µL of the cell suspension were mixed with 10 µL of phIL12 or pmIL12 (both at the concentration of 1 mg/mL), and the mixture was pipetted between stainless steel electrodes 2 mm apart followed by the delivery of electric pulses. The same electric pulse protocol (eight electric pulses, voltage-to-distance ratio of 1300 V/cm, pulse duration of 100 μs, frequency of 5 kHz) was applied as it will be used in the proposed exploratory clinical trial according to the updated standard operating procedure for the use of electrochemotherapy in the clinical settings [26,27,28]. Immediately after pulse delivery (<5 s), the mixture was transferred in a 24-well ultralow attachment plate (Corning, New York, NY, USA) [29]. Five min after GET, 1 mL of an appropriate cell culture medium was added, and the resulting suspension of the cells was transferred to an appropriate cell culture flask with a filter cap (T25 for 24 h and 48 h incubation; T75 for 72 h and 96 h incubation) and an additional cell culture medium was added. The cells were then grown in a humidified incubator at 37 °C and 5% CO_2_ until further processing.

**Figure 3:**
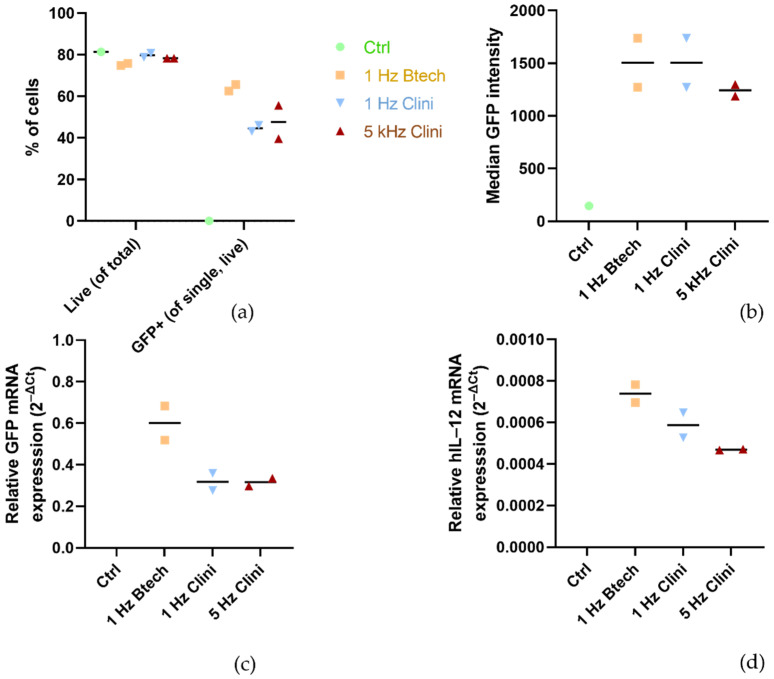
Efficacy of different electric pulse protocols. (**a**) Percentage of the live and GFP-positive cells; (**b**) median GFP intensity after GET of the pEGFP-N1 plasmid with different pulse protocols and devices. In all the three experimental groups (1 Hz Btech, 1 Hz Clini, 5 kHz Clini), eight 1300 V/cm pulses with 100 μs duration were used; only the frequency (1 Hz or 5 kHz) and the device used (BetaTech or Cliniporator^TM^) differed. The relative expression (normalized to β2-microglobulin) of transgene (**c**) GFP or (**d**) hIL-12 after different pulse parameters and devices was also tested 2 days after GET with qRT-PCR, as described further in Section 2.6.1. “Total RNA Isolation and cDNA Reaction” and Section 2.6.3 “qRT-PCR”.

An electrical pulse generator CLINIPORATOR^TM^ (IGEA S.p.A., Carpi, Italy) holding authorization for the use in the clinical environment for electrochemotherapy and gene therapy was used to deliver the pulses. The choice of the pulse parameter protocol was supported by publications describing gene therapy studies with similar plasmid coding for IL-12 performed in the US. The pulse protocol using six 100 μs pulses with the voltage-to-distance ratio of 1300 V/cm was used in published preclinical and clinical studies [11,30]. To our best knowledge, gene therapy studies with Cliniporator^TM^ and these specific parameters have not been published yet. Therefore, we performed some preliminary experiments shown in Figure 3 with plasmid pEGFP-N1 (BD Biosciences, Clontech, Palo Alto, CA, USA) as well as with phIL12 in the FaDu cells to confirm that the 5 kHz pulse protocol results in an adequate transfection efficiency compared to the 1 Hz protocol delivered by Cliniporator^TM^ and also compared to the 1 Hz protocol delivered by pulse generator BetaTech (LEROY Biotech, Saint-Orens-de-Gameville, France), the electroporation device that was also used in our preclinical studies [31,32]. In the clinical setting, the 5 kHz protocol is used for better patient’s compliance as it causes less discomfort to the patient than 1 Hz pulses.

### 2.5. Timeline of the Experiment and Sample Collection

The level and persistence of transgene expression was determined by means of quantitative real-time PCR (qRT-PCR) at the level of mRNA and using an enzyme-linked immunosorbent assay (ELISA) at the protein level [33,34]. As IL-12 is a secreted cytokine, the protein level was determined in the cell culture medium collected from the FaDu or CT26 cells after GET. To determine the kinetics of IL-12 expression after GET, the samples were collected 24, 48, 72 and 96 h after GET and, additionally, 7, 9, and 11 days after GET. Since the plasmid DNA copy number in the transfected cells rapidly decreased below the detection point, the copy number was determined only after 24, 48, 72 and 96 h after GET.

On the day of cell processing, the cell culture medium was collected, divided into aliquots and stored at −80 °C. One fraction was used for testing the biological activity and potency of phIL12 or pmIL12 and the other for quantification of the secreted hIL-12 or mIL-12 with an ELISA assay. The attached cells were counted and divided into two fractions; half of the cells were processed for quantification of transgene expression with qRT-PCR, the other half—for the determination of the copy number of plasmid DNA with qRT-PCR. The details of the abovementioned tests are given below.

### 2.6. Level of Transgene Expression and Plasmid Copy Number

#### 2.6.1. Total RNA Isolation and cDNA Reaction

In order to determine the level of transgene expression after GET, total RNA from the FaDu and CT26 cells was isolated. Isolation of total RNA was performed with a peqGOLD Total RNA Kit (VWR International, Radnor, PA, USA) according to the manufacturer’s instructions, including a DNA digestion step in order to digest the remaining plasmid DNA. The isolated RNA was stored at −80 °C until further use. Prior to reverse transcription into cDNA, the quantity and purity of the isolated RNA was determined using a Qubit 4 Fluorometer (Invitrogen, Thermo Fisher Scientific). Next, 1000 ng of the isolated RNA were reverse-transcribed into cDNA with a SuperScript VILO cDNA synthesis kit (Thermo Fisher Scientific) according to the manufacturer’s instructions. A thermal cycler (Primus 25 advanced^®^ Thermocycler, VWR) was used for reverse transcription with the following settings: incubation at 25 °C for 10 min, incubation at 42 °C for 60 min and termination of reaction at 85 °C for 5 min. The undiluted cDNA was stored at −80 °C until further use.

#### 2.6.2. Total DNA Isolation

In order to determine the plasmid copy number after GET, total DNA from the FaDu and CT26 cells was isolated. Isolation of total DNA was performed using a DNeasy Blood & Tissue Kit (Qiagen, Hilden, Germany). Before the isolation, the cells were resuspended in 2 mL of Hanks’ Balanced Salt Solution (HBSS) (with Mg^2+^ and Ca^2+^; Gibco), and plasmid DNA associated with the plasma membrane was digested with DNase I (168.1 U/µL, Invitrogen). The mixture was incubated at 37 °C for 10 min in Eppendorf ThermoMixer C (Eppendorf, Hamburg, Germany) at 300 rpm. After incubation, 200 µL of 0.5 M EDTA (Sigma-Aldrich) were added and slowly mixed by pipetting to deactivate DNase I. The cell suspension was then centrifuged for 5 min at 470× *g*. The supernatant was removed and the cells were resuspended in 200 µL of the PBS. The samples were then lysed using Proteinase K and RNA digested by adding RNase A (concentration, 7000 units/mL) (Qiagen). After 2 min incubation, a lysis buffer was added to the sample followed by 10 min incubation at 56 °C in Eppendorf Thermomixer C at 300 rpm. When incubation was finished, 200 µL of ethanol were added and thoroughly vortexed. The mixture was pipetted to a DNeasy Mini spin column and centrifuged for 1 min at 6500× *g*. The DNeasy Mini spin column was placed into a new collection tube and 500 µL AW1 buffer was added following centrifugation at 6500× *g* for 1 min. The DNeasy Mini spin column was transferred to a new collection tube and 500 µL of the AW2 buffer were added and centrifugation at 20,000× *g* was performed for 3 min. The DNeasy Mini spin column was transferred to a new 1.5 mL tube and 50 µL of the elution buffer were added following 1 min incubation at room temperature. After incubation, centrifugation at 6500× *g* was performed for 1 min. The last step was repeated once. The isolated DNA was stored at −80 °C until further use. The quantity and purity of the isolated DNA was determined with a Qubit 4 Fluorometer.

#### 2.6.3. qRT-PCR

For the qRT-PCR assay, SYBR Green chemistry was used, which enables relative as well as absolute quantification of the target sequence in the investigated sample. Relative quantification was used to determine the expression level of the transgene in the FaDu and CT26 cells after GET where the untreated cells were used as a reference sample. We designed the primers specific for our transgene that would not amplify endogenous IL-12 mRNA of either human or murine origin (Integrated DNA Technologies; IDT, Newark, NJ, USA; primer details are in Table 1) or any other known double-stranded (ds) DNA sequence. Additionally, absolute quantification was used to determine the plasmid copy number in both cell lines after GET, where the standard curve method was used to calculate the plasmid copy number based on the serial dilution series of synthetic dsDNA (gBlocks, IDT). For this part, we designed a different set of primers specific for the phIL12 plasmid and the pmIL12 plasmid that do not amplify any other known dsDNA sequence (primer details are in Table 1). No template control (NTC) including all the PCR reagents with the exception of the template (cDNA) was used as the negative control for the qRT-PCR reaction. All the samples were run in technical duplicates. As the internal/housekeeping control in the samples isolated from the murine CT26 cells, β-actin (IDT) was used, while the expression of the β-glucuronidase (GUSB, IDT) gene was followed in the samples isolated from the human FaDu cells (primer details in Table 1). For the experiments evaluating the pulse parameter protocols (Figure 3c,d), the relative expression of transgene GFP or hIL-12 was normalized to β2-microglobulin (primer details in Table 1).

For each qRT-PCR reaction, 20 µL of the reaction mixture containing 10 ng of the cDNA or 10, 1 or 0.1 ng of total DNA was used. The working concentration of the primers was 200 nM. A PowerUP^TM^ SYBR^TM^ Green Master Mix (2×) (Thermo Fisher Scientific) was used according to the manufacturer’s instructions. The reactions were run in 96-well PCR plates on QuantStudio 3 (Thermo Fisher Scientific). The thermal cycling conditions for the determination of gene expression levels were as follows: 2 min at 50 °C, 2 min at 95 °C, 40 cycles of 15 s at 95 °C, 1 min at 60 °C; for the melting curve determination, 15 s at 95 °C, 1 min at 60 °C, 15 s at 95 °C. The thermal cycling conditions for the determination of the plasmid copy number were as follows: 2 min at 50 °C, 2 min at 95 °C, 40 cycles of 15 s at 95 °C, 30 s at 58 °C and 30 s at 72 °C; for the melting curve determination, 15 s at 95 °C, 1 min at 60 °C, 15 s at 95 °C.

After the qRT-PCR run, the data were analyzed with the QuantStudio’s software. The Ct values were determined for each primer set and each sample. Relative quantification of transgene expression was expressed as the relative quantity of the transgene (IL-12) compared to the quantity of the housekeeping gene. Absolute quantification of the plasmid copy number in the cells after GET was based on the serial dilution series of gBlocks, which was used to determine the standard curve.

### 2.7. ELISA Assay

For the ELISA assay (Human IL-12 p70 Quantikine ELISA Kit, R&D Systems, Minneapolis, MN, USA, or Mouse IL-12 p70 Quantikine ELISA Kit, R&D), the cell culture medium from the FaDu and CT26 cells was collected at different timepoints after GET designated in Figure 1. The cell culture medium was centrifuged and the supernatant was then divided to aliquots and stored at −80 °C. All the samples, standards and controls were assayed in duplicate. The ELISA assay was performed according to the manufacturer’s instructions. Within 30 min after the end of the assay, optical density (OD) for each well was measured using a spectrophotometer (Cytation 1, BioTek Instruments) at 450 and 570 nm. The reading value at 570 nm was subtracted from the 450 nm value to correct for optical imperfections in the plate. The duplicate readings for each standard, control and sample were averaged and then the average zero standard optical density (OD) was subtracted from them. The standard curve was created by reducing the data to generate a four-parameter logistic (4-PL) curve fit according to the manufacturer’s instructions. The obtained equation was then used to calculate the concentration of hIL-12 p70 or mIL-12 p70 in the samples. For each sample, the total concentration of the protein in the cell media was indicated as the average OD value × volume of cell media/number of cells.

### 2.8. Biological Activity and Potency

The cell culture medium was collected one day after GET to determine the biological activity and potency of the transgene (secreted IL-12) in both the FaDu and CT26 cell lines. Biological activity and potency were additionally confirmed 48 h after the transfection of target cells with lipofection. One day before lipofection, 0.85 × 10^5^ FaDu or 0.5 × 10^5^ CT26 cells were seeded per each well in a 24-well plate. On the day of transfection, 1 µL of Lipofectamine 3000 (Thermo Fisher Scientific) was diluted in 25 µL Opti-MEM medium (Thermo Fisher Scientific). In a separate tube, the mixture of 0.5 µL of plasmid DNA at a concentration of 1 mg/mL, 25 µL Opti-MEM medium and 1 µL of the P3000 reagent was prepared. Next, the dilution of plasmid DNA and the dilution of Lipofectamine 3000 were combined at a 1:1 ratio. The mixture was gently mixed and incubated for 15 min at room temperature. After incubation, 50 µL of the mixture were transferred to the cells; 48 h after the transfection, the cell media were removed to determine the biological activity and potency of pmIL12 and phIL12.

The biological activity and potency were tested using HEK-Blue™ IL-12 cells, which were designed to detect bioactive human and murine IL-12 by monitoring the activation of the STAT4 pathway through the detection of produced secreted alkaline phosphatase (SEAP). SEAP in the supernatant of the HEK-Blue™ IL-12 cells was detected using a Phospha-Light™ SEAP Reporter Gene Assay System (Thermo Fisher Scientific). Briefly, a suspension of HEK-Blue™ IL-12 was prepared in a prewarmed growth medium with 1× HEK-Blue™ Selection (InvivoGen) at a concentration of 280,000 cells/mL. The HEK-Blue™ IL-12 cell suspension (180 µL (~50,000 cells)) was added to each well in a 96-well plate. The cell culture medium (20 µL) collected after GET was added to the cell suspension. In separate wells, 20 µL of the recombinant human IL-12 standard (WHO Reference Reagent Interleukin 12, National Institute for Biological Standards and Control, Hertfordshire, UK) prepared in different dilutions were added to the cell suspension in order to construct a standard curve. The plates were incubated overnight at 37 °C in 5% CO_2_. The next day, a Phospha-Light™ SEAP Reporter Gene Assay System (Thermo Fisher Scientific) was used according to the manufacturer’s instructions. The emitted luminescence was measured with spectrophotometer Cytation 1, and the biological activity/potency of the transgene was expressed as “equal to the quantity of recombinant hIL-12 in international units (IU)” extrapolated from the constructed standard curve obtained from the WHO standard.

### 2.9. Statistical Analysis

For statistical analysis and graphical representation, GraphPad Prism 9 (GraphPad software, San Diego, CA, USA) was used. Since the data were normally distributed, the significance was determined using the two-tailed *t*-test or one-way ANOVA test; *p* < 0.05 was considered statistically significant.

## 3. Results

### 3.1. Production Process

**Figure 4:**
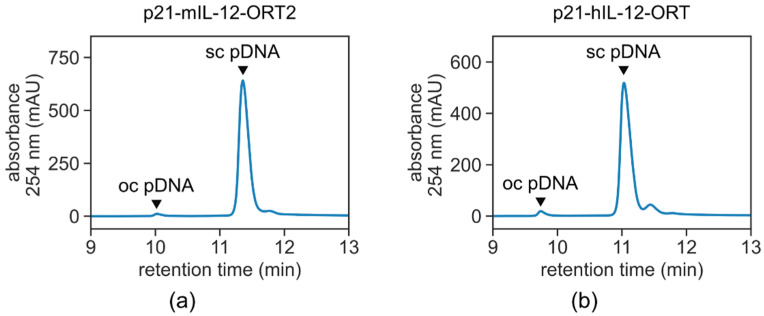
HPLC analytics of bulk products. Conditions: CIMac™ pDNA-0.3 analytical column; flow rate of 1 mL/min; mobile phase (**a**): 200 mM Tris, pH 8.0; mobile phase (**b**): 200 mM Tris, 1.0 M NaCl, pH 8.0; UV detection—260 nm.

Several production processes were run to prepare a sufficient amount of plasmid DNA for in vitro and in vivo non-clinical trials. For the p21-hIL12-ORT production, two fed-batch fermentations were processed, resulting in 31 ± 3 g of dry cell weight (DCW) per liter containing 21.3 ± 9.5 mg of plasmid DNA/L fermentation broth. The purified plasmid from both batches was pooled and the sterile final product was prepared with the final concentration of 1.93 mg/mL. In total, 9.6 mg of plasmid p21-hIL12-ORT were prepared for in vitro studies. The drug product was characterized using the established methods (Figure 4, Table 2).

For the p21-mIL12-ORT production, more batches were required in order to test process robustness and produce drug products for both in vitro and in vivo studies. Three complete productions from fed-batch fermentation to final product formulation were performed. In order to achieve higher purity in terms of HCP impurities, an additional run of diafiltration was implemented before sterile filtration of the final drug product which resulted in an additional 10-fold decrease in the HCP content compared to the final p21-hIL12-ORT product. Robustness of the production process for clinical grade plasmid production can be recognized by comparing fermentation yields and drug product characterization: fermentation processes resulted in 28 ± 6 g of DCW per liter containing 10.0 ± 3.1 mg of plasmid DNA/L fermentation broth. The final product characterization resulted in 98.3 ± 1.1% Sc isoform, 12 ± 3 ng gDNA/mg pDNA, 0.6 ± 0.2 µg HCP/mg pDNA, 0.1 ± 0.2 EU/mg pDNA. Three lots of the plasmid resulted in the production of 38.6 mg total plasmid mass with the final concentration of 1.95 mg/mL ± 0.04. However, only one lot was used for in vitro studies (Figure 4, Table 2).

### 3.2. GET of phIL12 in the FaDu Cell Line

**Figure 5:**
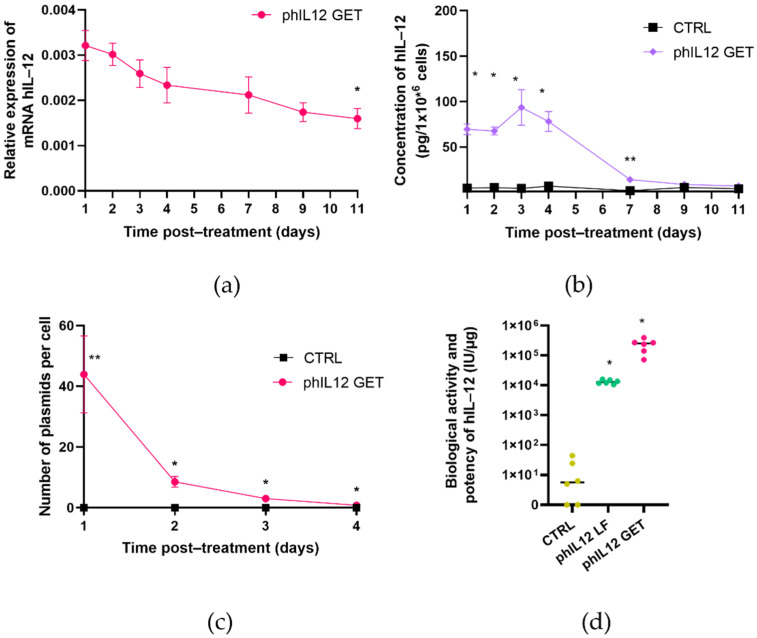
GET of phIL12 in the FaDu cell line. (**a**) Relative expression of hIL-12 mRNA in the target cells; * *p* < 0.05—statistically significant difference compared to day 1. (**b**) Expression of hIL-12 on the protein level; * *p* < 0.05—statistically significant difference compared to the expression levels at days 7, 9 and 11 and the control group; ** *p* < 0.005—statistically significant difference compared to the expression levels at days 1, 2, 3 and 4 and the control group. (**c**) Copy number of plasmid DNA per cell; ** *p* < 0.005—statistically significant difference compared to the copy number at days 2, 3 and 4 and the control group; * *p* < 0.05—statistically significant difference compared to the copy number at day 1 and the control group. (**d**) Biological activity and potency of the hIL-12 protein in control untreated cells (dark yellow), in phIL12 Lipofectamine (LF) transfected cells (green) and in phIL12 gene electrotransfer (GET) transfected cells (pink); * *p* < 0.05—statistically significant difference compared to the control cells. All the experiments were repeated three times, each of them including two separate biological replications.

GET of phIL12 resulted in the production of biologically active IL-12 in the FaDu cells. The expression of human IL-12 was confirmed both at the mRNA and protein levels. Briefly, the IL-12 mRNA expression reached the maximum 1 day after GET and steadily decreased with time (Figure 5a). Expression of the transgene IL-12 mRNA in the control samples was not detected. Protein expression showed maximal increase in the hIL-12 levels in the first 4 days post-treatment, followed by the decrease until day 9 until it reached the control values (Figure 5b). Minimal amounts of the IL-12 protein in the control cells were detected due to endogenous IL-12. GET of phIL12 resulted in the average of 44 copies of plasmid DNA per cell detected 1 day post-treatment. The DNA plasmid copy number exponentially decreased with time in the observed 4-day period (Figure 5c). The biological activity and potency of hIL-12 after phIL12 GET was determined using HEK-Blue™ IL-12 cells. Secreted human IL-12 in the cell media of the transfected FaDu cells successfully activated the STAT4 pathway, leading to increased SEAP production compared to the control group. The measured biological activity of hIL-12 was 2.3 ± 0.5 × 10^5^ IU/µg. The biological activity and potency of hIL-12 expressed from plasmid phIL12 were additionally confirmed after lipofection for every batch of phIL12 used in the experiments and was 1.3 ± 0.9 × 10^4^ IU/µg (Figure 5d). The values of biological activity of the produced proteins are in the range of commercially available hIL-12 recombinant proteins (from 1 × 10^3^ to >2 × 10^4^ IU/µg) [35,36].

### 3.3. GET of pmIL12 in the CT26 Cell Line

**Figure 6:**
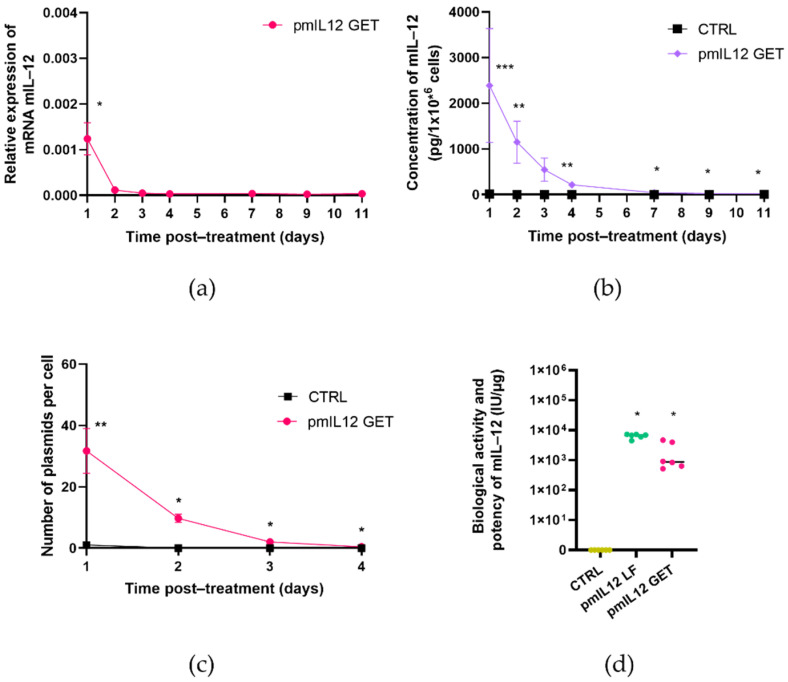
GET of pmIL12 in the CT26 cell line. (**a**) Relative expression of mIL-12 mRNA in the target cells; * *p* < 0.0001—statistically significant difference compared to all the other timepoints. (**b**) Expression of mIL-12 on the protein level; * *p* < 0.05—statistically significant difference compared to the protein concentration at day 1 and the control; ** *p* < 0.05—statistically significant difference compared to the control; *** *p* < 0.05—statistically significant difference compared to the protein concentration at days 7, 9 and 11. (**c**) Copy number of plasmid DNA per cell; ** *p* < 0.005—statistically significant difference compared to the copy number at days 2, 3 and 4 and the control; * *p* < 0.005—statistically significant difference compared to the copy number at day 1 and the control. (**d**) Biological activity and potency of the mIL-12 protein in control untreated cells (dark yellow), in pmIL12 Lipofectamine (LF) transfected cells (green) and in pmIL12 gene electrotransfer (GET) transfected cells (pink); * *p* < 0.05—statistically significant difference compared to the control cells. All the experiments were repeated three times, each of them including two separate biological replications.

Similarly to phIL12, GET of pmIL12 resulted in the production of biologically active murine IL-12 in the CT26 cells. Its expression was confirmed both at the mRNA and protein levels, with slightly different expression dynamics compared to the expression of human IL-12 in the FaDu cells. The relative murine mRNA levels reached the maximum 1 day after GET and then rapidly decreased until day 4 post-treatment (Figure 6a). The expression of IL-12 mRNA in the control samples was not detected. Protein expression showed maximal increase in the mIL-12 levels already 1 day post-treatment, followed by a steady decrease in the protein levels until day 11 (Figure 6b). GET of pmIL12 resulted in the average of 32 copies of plasmid DNA per cell, detected 24 h post-treatment (Figure 6c). Similarly to phIL12, the pmIL12 DNA plasmid copy number exponentially decreased in the observed 4-day period. The biological activity and potency of secreted murine IL-12 were confirmed 24 h after GET as well after lipofection for every batch of pmIL12 used in the experiments. The biological activity of murine IL-12 was 1.9 ± 0.8 × 10^3^ IU/µg after GET and 6.4 ± 0.4 × 10^3^ IU/µg after lipofection (Figure 6d).

## 4. Discussion

Gene electrotransfer of the plasmid encoding IL-12 (GET IL-12) holds a great potential in cancer immunotherapy. This application is already in clinical trials in the USA, demonstrating encouraging results in the treatment of melanoma patients [37]. Intratumoral GET IL-12 has proven safe and efficient, having good local tumor control, and some evidence indicates abscopal effect. To comply with the EU regulatory requirements for clinical application, in our previous study, we constructed plasmids devoid of the antibiotic resistance gene encoding human or murine IL-12 under the transcriptional control of the p21 promoter. This promoter was proven useful for controlling transgene expression in a tumor-specific manner and is inducible by genotoxic stress [15,38], making it attractive for combination with cancer treatments that are known to induce genotoxic stress, such as radiotherapy or electrochemotherapy. In this combination, local ablative treatment could act as in situ vaccination, releasing tumor antigens from the therapy- killed tumor cells, and IL-12 GET as an immunological adjuvant, boosting the primed immune response [39]. 

Moreover, to comply with the guideline on quality, non-clinical and clinical requirements for investigational advanced therapy medicinal products in clinical trials, we had to prepare a murine ortholog of plasmid phIL12 since human IL-12 is not active in mice. Plasmid pmIL12 encoding murine IL-12 was thus constructed.

A manufacturing process compliant with the EMA, the USP and the ICH was developed and optimized for both plasmids. The process is amenable for scale-up and cGMP production. The plasmids produced are of high quality, meeting all the required specifications for plasmid identity, quality and purity. Fermentation process yields resulted in less than 30 mg pDNA/L fermentation broth which is low compared to the established plasmid production platforms. Different plasmid production yields were reported, ranging from 100–250 mg pDNA/L fermentation broth [21] to as high as 2600 mg pDNA/L [40]. A vast set of parameters influence fermentation yields which can be divided in two main groups: fermentation strain design and process-specific parameters, both of them exploited for plasmid production optimization [40,41].

Both plasmids were first evaluated in vitro in CT26 and FaDu cell lines in compliance with the guidelines and directives for non-clinical evaluation of gene therapy products. The in vitro evaluation indicated that GET of phIL12 and pmIL12 resulted in the production of biologically active IL-12 in the FaDu and CT26 cells, respectively. Increased mRNA levels of mIL-12 and hIL-12 in the transfected cells were detected together with the increased concentration of secreted IL-12 in cell media. Importantly, although only 0.025‰ of the initially added plasmid (10 μg) was transferred into the cells, this number of plasmids was sufficient to expect efficient transgene expression and a notable effect in further in vivo studies in murine models [42]. Furthermore, the expression could be further increased by adding a stress stimulus such as irradiation. Based on our previously published data [14], we believe that a maximum of twofold increase in IL-12 expression could potentially be achieved if the tumors are irradiated after GET of phIL12.

Importantly, the transgene transcription level as well as the concentration of biologically active proteins and the plasmid copy number after GET showed a time-dependent decrease. Indeed, the kinetics of the expression at the mRNA and protein levels between phIL12 and pmIL12 differed slightly, which is not surprising for the transfection of two different cell lines. As is well-known from the literature [43], differences in transgene expression after GET could be assigned to the differences in cell size and shape, doubling time and other biological characteristics of the electroporated cells.

The aim of in vitro non-clinical evaluation of gene therapy product phIL12 and its ortholog pmIL12 was to confirm the expression and biological activity of the expressed transgenes for further in vivo non-clinical studies. The final goal was to implement GET of phIl12 in the first-in-human clinical trial, evaluating the safety and tolerability of the constructed plasmid for the treatment of basal cell carcinomas in patients. The results of the trial will provide valuable data, including for other future and ongoing studies involving GET IL-12. The perspective of GET IL-12 in cancer therapy lies mostly in combination approaches combining GET IL-12 with other conventional or experimental cancer treatments [1]. One such potential approach is the use of GET IL-12 in combination with radiotherapy. A preclinical study of combined GET of the plasmid encoding mIL-12 and radiotherapy demonstrated increased intratumoral cytokine levels. In murine sarcoma tumors, an increased complete response rate with no significant irradiation-induced damage to the normal tissue was observed after combined treatment [44]. A good antitumor effect was also demonstrated in a murine adenocarcinoma tumor model in a study using GET of the p21 promoter-driven IL-12 plasmid in combination with local tumor irradiation, which was comparable to the same treatment using a constitutive promoter [15]. Another study on sarcoma models proved the effectiveness of systemic IL-12 GET administered into the cranial tibial muscle in combination with irradiation of tumors and lung metastases [45].

Another potential approach is the combination of GET IL-12 with the administration of chemotherapeutics. We recently proposed a model of in situ vaccination [39] that includes electrochemotherapy (ECT) boosted with GET IL-12. Unlike the conventional cancer vaccines, in situ vaccination is based on the activation of the immune system against the endogenous tumor antigens. In the proposed combination, it was demonstrated how ECT acts as an in situ vaccine, causing the release of many tumor antigens (TAA) present at the tumor site, and how IL-12 GET as an immunological adjuvant boosted the primed immune response against the TAA released from the killed tumor cells [39,46,47,48]. The feasibility of the model was demonstrated in a clinical trial on client-owned dogs with mast cell tumors, where the combination of electrochemotherapy with IL-12 gene therapy resulted in a high percentage of cured tumor and prevented recurrence and development of distant metastases [49,50,51]. The same effect of in situ vaccination is also expected in the combination of GET IL-12 with other local therapies such as radiation [44] and other ablative techniques [1].

In addition to local therapies combined with GET IL-12, combinations of GET IL-12 with immune checkpoint inhibitors are under investigation. Due to the profound intratumoral T cell infiltration induced by GET IL-12, the treatment can be expected to synergize with checkpoint inhibitors, particularly to enhance their efficiency against “cold” tumors [1]. Currently, there are several studies ongoing in the USA for the treatment of melanoma, breast cancer and head and neck tumors in combination with immune checkpoint inhibitors (ClinicalTrials.gov). Lastly, the plasmid encoding IL-12 could also be used as an adjuvant to enhance the efficiency of DNA vaccines for different diseases. The efficiency of IL-12 as an immunogenic adjuvant has already been evaluated and confirmed in preclinical studies in different animal models investigating the effectiveness of HIV/SIV or hepatitis C vaccines [52,53].

## 5. Conclusions

In compliance with the EU guidelines on quality, non-clinical and clinical requirements for investigational advanced therapy medicinal products in clinical trials, antibiotic resistance gene-free plasmids encoding human or murine IL-12 were constructed and evaluated in vitro. A scalable production process for clinical grade plasmid manufacturing with relevant analytical methods was established, and thoroughly characterized plasmids were produced for non-clinical evaluation. The process is amenable to scale-up and cGMP manufacturing. In the described study, GET of phIL12 and pmIL12 was proven as an efficient delivery method resulting in the production of biologically active IL-12 in the FaDu and CT26 cells, respectively. The transgene transcription level as well as the biologically active protein and plasmid copy number after GET showed a time-dependent decrease. Thus, our results demonstrate that both constructed plasmids are suitable for further in vivo studies in murine models. Such non-clinical evaluation sets the basis for further evaluation of gene therapy product phIl12 in human clinical trials. Detailed evaluation of GET IL-12 would therefore contribute to easier translation of the approach in clinic not only as a monotherapy, but also in combination with other local or systemic cancer treatments.

## Figures and Tables

**Table 1:** Details of the primers used for qRT-PCR.

Primer	Primer Details	Sequence
hIL-12, forward	Expression primer: specific for the linker region between the p40 and p35 IL-12 subunits (phIL12)	CTGCAGTGTTCCTGGAGTAG
hIL-12, reverse	Expression primer: specific for the linker region between the p40 and p35 IL-12 subunits (phIL12)	GAACATTCCTGGGTCTGGAG
mIL-12, forward	Expression primer: specific for the linker region between the p40 and p35 IL-12 subunits (pmIL12)	CCGATCGGTTCCTGGAGTA
mIL-12, reverse	Expression primer: specific for the linker region between the p40 and p35 IL-12 subunits (pmIL12)	GGGACTGGCTAAGACACCT
phIL12 and pmIL12 copy number, forward	Copy number primer: specific for the plasmid backbone (ori)	GCAGAGCGCAGATACCAAATA
phIL12 in pmIL12 copy number, reverse	Copy number primer: specific for the plasmid backbone (ori)	GCGCCTTATCCGGTAACTATC
hGUSB, forward	Human internal/housekeeping expression control	AGGTGATGGAAGAAGTGGTG
hGUSB, reverse	Human internal/housekeeping expression control	AGGATTTGGTGTGAGCGATC
mβ-actin, forward	Murine internal/housekeeping expression control	CTGTGCTGTCCCTGTATGC
mβ-actin, reverse	Murine internal/housekeeping expression control	GGCACAGTGTGGGTGAC
Synthetic dsDNA (gBlocks)		GTAACTGGCTTCAGCAGAGCGCAGATACCAAATACTGTTCTTCTAGTGTAGCCGTAGTTAGGCCACCACTTCAAGAACTC TGTAGCACCGCCTACATACCTCGCTCT GCTAATCCTGTTACCAGTGGCTGCTGCCAGTGGCGATAAGTCGTGTCTTACCGGGTTGGACTCAAGACGATAGTTACCGGATAAGGCGCAG CGGTCGGGCTGAACGG-GGGGTTC
GFP	Predesigned expression primer for GFP	Mr04097229_mr, Thermo Fisher Scientific
hβ2-microglobulin, forward	Human internal/housekeeping expression control	GGCATTCCTGAAGCTGACAG
hβ2-microglobulin, reverse	Human internal/housekeeping expression control	TGGATGACGTGAGTAAACCTG

**Table 2:** Specification of plasmid DNA used for in vitro studies.

		Method	Specification	p21-hIL-12-ORT	p21-mIL-12-ORT
pDNA	Oc	HPLC	<3%	2.4%	2.56%
	Ln		<3%	0.16%	0.43%
	Sc		>94%	97.47%	97.00%
pDNA concentration		A 260	1.8–2.0 mg/mL	1.93 mg/mL	1.99 mg/mL
RNA		AGE	Not visible on agarose gel	Not visible on agarose gel	Not visible on agarose gel
gDNA		qPCR	<20 μg/mg pDNA	11.8 μg/mg pDNA	10.4 μg/mg pDNA
Host cell proteins		microBCA	<3 μg/mg pDNA	7.0 μg/mg pDNA	0.72 μg/mg pDNA
Endotoxin		According to Ph. Eur. 2.6.14., USP <85>	<10 EU/mg pDNA	2.9 EU/mg pDNA	0.03 EU/mg pDNA
Sterility		According to Ph. Eur. 2.6.1., USP <71>	Negative	Negative	Negative
pH		pH-meter	5.8–6.3	6.3	5.9
Appearance		According to Ph. Eur. 2.9.20.	Clear/transparent solution	Clear/transparent solution	Clear/transparent solution

AGE, agarose gel electrophoresis; gDNA, genomic DNA, HPLC, high-performance liquid chromatography; Ln, linear; Oc, open circular; Ph. Eur., European Pharmacopoeia; EU, endotoxin unit; Sc, supercoiled; USP, United States Pharmacopeia.

## Data Availability

The data presented in this study are available on request from the corresponding author.

## Supplementary Materials

The following are available online at https://www.mdpi.com/article/10.3390/pharmaceutics13101739/s1, Figure S1: Selection of clones containing antibiotic resistance gene-free version of p21-mIL-12-ORT plasmid. (a) Clone 29 (in bold) was selected for further analysis based on miniprep isolation yields, restriction analysis and presence of supercoiled monomer band. Minipreps were performed form 3 ml of overnight single colony culture by GeneJET Plasmid Miniprep Kit (Thermo Fisher Scientific). (b) Representative example of electrophoretic evaluation of clones 23, 39, 54 and 29. Isolated plasmids from clones 23, 39, 54 and 29 were loaded onto a electrophoretic gel either cut with ApaLI and KpnI restriction enzymes or uncut and their identity was confirmed by positive matching of the band pattern on the electrophoresis gel to the expected pattern obtained by a simulation experiment using SnapGene software. For the uncut plasmids, the simulated band pattern differs from the actual pattern, because simulation can only be done for supercoiled monomer, while other forms (supercoiled dimer, open circular, linear, nicked) can also be seen on the electrophoretic gel. Electrophoresis details: 1% agarose (Sigma Aldrich), run 45 min at 100 V/cm, stained in 1xSybrGold. LL (Linear DNA ladder): GeneRuler™ 1 kb Plus DNA Ladder (Thermo Fisher Scientific), SC (Supercoiled DNA Ladder): Supercoiled DNA Ladder (New England BioLabs).
