# Supplementary material for: Non-Clinical In Vitro Evaluation of Antibiotic Resistance Gene-Free Plasmids Encoding Human or Murine IL-12 Intended for First-in-Human Clinical Study
Source: Pharmaceutics. 2021 Oct 19;13(10):1739. doi: 10.3390/pharmaceutics13101739 (PMC8539770; doi:10.3390/pharmaceutics13101739)
Supplement: Supplementary file 1 [file pharmaceutics-13-01739-s001.zip › pharmaceutics-1390810 -supplementary.pdf]

pharmaceutics

MDPI

Supplementary Materials: Non-Clinical In Vitro Evaluation of Antibiotic Resistance Gene-Free Plasmids Encoding Human or Murine IL-12 Intended for First-in-Human Clinical Study

Spela Kos, Masa Bosnjak, Tanja Jesenko, Bostjan Markelc, Urska Kamensek, Katarina Znidar, Urska Matkovic, Andrej Rencelj, Gregor Sersa, Rosana Hudej, Aneja Tuljak, Matjaz Peterka and Maja Cemazar

Clone No	Isolation yield (ng/µl)	Restriction analysis	Supercoiled monomer
7	330	×	×
11	170	×	×
13	80	✓	✓
23	380	✓	×
**29**	**280**	✓	✓
32	280	×	×
39	70	✓	✓
54	500	×	×
55	210	✓	×
57	220	✓	✓
60	170	✓	✓

(a)

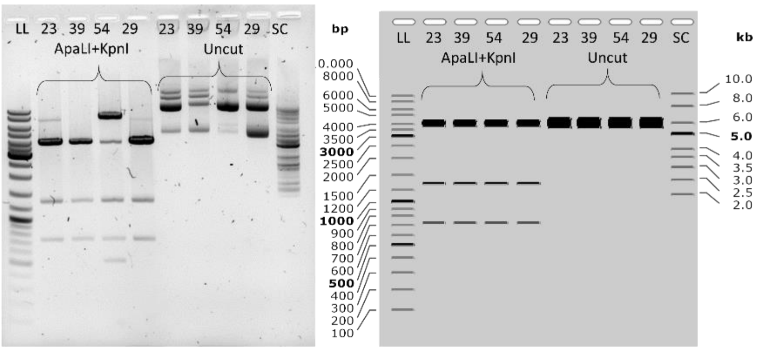

(b) (c)

Supplementary Figure S1. Selection of clones containing the antibiotic resistance gene-free version of the p21-mIL-12-ORT plasmid. (**a**) Clone 29 (in bold) was selected for further analysis based on the miniprep isolation yields, the restriction analysis and the presence of a supercoiled monomer band. Minipreps were performed from 3 mL of the overnight single colony culture using a GeneJET Plasmid Miniprep Kit (Thermo Fisher Scientific). (**b**) Representative example of electrophoretic evaluation of clones 23, 39, 54 and 29. Isolated plasmids from clones 23, 39, 54 and 29 were loaded onto an electrophoretic gel either cut with the *ApaL*I and *Kpn*I restriction enzymes or uncut and their identity was confirmed by positive matching of the band pattern on the electrophoresis gel to the expected pattern obtained in a simulation experiment using the SnapGene software. For the uncut plasmids, the simulated band pattern differs from the actual pattern because simulation can only be done for a supercoiled monomer, while other forms (supercoiled dimer, open circular, linear, nicked) can also be seen on the electrophoretic gel. Electrophoresis details: 1% agarose (Sigma-Aldrich), run for 45 min at 100 V/cm, stained in 1× Sybr Gold. LL (linear DNA ladder): GeneRuler™ 1 kb Plus DNA Ladder (Thermo Fisher Scientific), SC (supercoiled DNA ladder): Supercoiled DNA Ladder (New England BioLabs).
